# Job insecurity and work passion among Central Sterile Supply Department nurses in the post-COVID-19 context

**DOI:** 10.3389/fpubh.2026.1769574

**Published:** 2026-05-19

**Authors:** Yan Zhang, Xixi Cai

**Affiliations:** Central Sterile Supply Department, The Third People's Hospital of Chengdu, Chengdu, Sichuan, China

**Keywords:** Central Sterile Supply Department nurses, COVID-19, job insecurity, organizational identification, work passion

## Abstract

Central Sterile Supply Department (CSSD) nurses played a critical role during the COVID-19 pandemic, managing the collection, cleaning, disinfection, sterilization, and distribution of medical instruments. Their workload and occupational risk increased sharply. However, research on the relationship between CSSD nurses’ job insecurity and work passion, as well as the underlying mechanisms, remains scarce, especially from an individual-centered perspective that explores psychological heterogeneity within this nurse population. To address these gaps, we recruited 496 CSSD nurses from 26 secondary and tertiary hospitals across Sichuan, China, between September and October 2025. The survey included scales measuring job insecurity, work passion, organizational identification, and demographic variables. Structural equation modeling was used to examine the mediating role of organizational identification, while latent profile analysis identified subgroups based on job insecurity and organizational identification, and compared differences in work passion across these profiles. Results revealed a significant negative correlation between job insecurity and work passion among CSSD nurses in the post-COVID-19 context, with organizational identification partially mediating this relationship. Latent profile analysis identified three distinct subgroups: “High Job Insecurity–Low Organizational Identification,” “Moderate Job Insecurity–Moderate Organizational Identification,” and “Low Job Insecurity–High Organizational Identification,” with the latter exhibiting the highest levels of work passion. This study elucidates the dual-perspective mechanism linking job insecurity and work passion among CSSD nurses in the post-COVID-19 context. The findings provide empirical support for hospital administrators to develop stratified, targeted psychological support strategies, emphasizing the enhancement of organizational identification to buffer the negative impact of job insecurity on nurses’ work passion.

## 1 Introduction

Since the outbreak of COVID-19 in late 2019, global public health systems have faced unprecedented challenges, drawing widespread attention to healthcare workers’ occupational conditions and psychological well-being (1, 2). Nurses were exposed to prolonged workloads, infection risks, intensive protective requirements, and rapidly changing care procedures, which increased both physiological exhaustion and psychological strain. Previous studies have reported that hospital nurses experienced elevated psychological distress, poorer professional quality of life, effort–reward imbalance, and stronger turnover intention during the pandemic, indicating substantial impairment in occupational well-being (3). In addition, effort–reward imbalance and burnout have been shown to negatively influence compliance with standard precautions among nurses and midwives, suggesting that sustained occupational stress may impair not only mental health but also safety-related work behavior (4). Even in the post-pandemic period, healthcare organizations may continue to face residual uncertainty related to infection prevention and control requirements, staffing arrangements, and workflow adjustments (5, 6). Among healthcare personnel, Central Sterile Supply Department (CSSD) nurses serve as a core component of hospital infection control, experiencing a marked increase in workload and occupational hazards during this period (7, 8). CSSD nurses are responsible for the critical processes of collecting, cleaning, disinfecting, sterilizing, and distributing medical instruments (9, 10). Their frequent exposure to potentially pathogenic microorganisms and high-intensity chemical agents (11), combined with extended shifts in highly protective environments, subjects them to prolonged high-risk and high-stress conditions. Consequently, CSSD nurses are particularly vulnerable to experiencing job insecurity—the subjective apprehension regarding future job stability and personal safety (12, 13)—in the post–COVID-19 context, where enhanced infection-control requirements and workflow adjustments may persist. Existing literature confirms that COVID-19 and its aftermath has significantly altered nurses’ psychological structures and organizational behaviors (14, 15); yet, there is a notable research gap regarding CSSD nurses specifically. This group differs from frontline clinical nurses, who face direct disease exposure, and from lower-risk auxiliary staff. Their unique occupational profile suggests distinct psychological states and organizational identification dynamics, warranting focused investigation. Therefore, this study prioritizes exploring CSSD nurses’ job insecurity, given its critical research value.

Job insecurity refers to individuals’ subjective concerns about the continuity of their employment and the stability of their work environment (12, 16). It stems not only from external structural changes but also from internal psychological resources and perceptions of organizational support (17, 18). Against the complex backdrop of COVID-19, nurses face multiple uncertainties, including increased workload, insufficient protective measures, and institutional adjustments, continuously challenging their psychological safety boundaries (19, 20). Prolonged job insecurity may trigger anxiety, emotional exhaustion, burnout, and reduced professional commitment, thereby undermining employees’ positive work engagement and passion for work (21, 22). In parallel, persistent effort–reward imbalance may further intensify perceptions of unfairness and resource depletion, making it more difficult for nurses to sustain enthusiasm, meaning, and intrinsic motivation in their work. From this perspective, work passion may be particularly vulnerable in high-risk care environments where uncertainty, heavy demands, and insufficient psychosocial returns coexist. In this context, work passion, defined as a highly affective, intrinsically motivated psychological state toward one’s work (23, 24), emerges as a vital variable to understand nurses’ psychological adaptation and occupational behavior in crisis settings. Work passion encompasses not only positive emotional experiences but also the integration of cognitive and behavioral resources that enable individuals to maintain engagement, growth, and creativity under pressure or uncertainty (25, 26). However, persistent occupational stress and external threats during the pandemic may diminish nurses’ enthusiasm and sense of professional value (27, 28). In addition to job-related uncertainty, nurses in the COVID-19 and post-COVID-19 context have often faced broader occupational strain characterized by burnout, effort–reward imbalance, emotional exhaustion, and reduced professional well-being. Burnout, typically manifested through emotional exhaustion, depersonalization, and reduced personal accomplishment, has become a major concern in nursing populations exposed to prolonged high-intensity work demands. Likewise, effort–reward imbalance reflects a mismatch between the substantial physical and psychological effort invested by nurses and the rewards they receive in return, such as recognition, income, career security, and organizational support. These conditions are highly relevant to CSSD nurses, whose work is essential to infection prevention and control but often receives limited visibility and recognition within hospital systems. Therefore, examining job insecurity among CSSD nurses should be situated within a broader framework of occupational stress and motivational resource depletion. Still, some nurses demonstrate high work passion and mission-driven motivation (29), suggesting that even after the acute phase of COVID-19, the association between job insecurity and work passion may be complex and shaped by multifaceted organizational and individual factors. Accordingly, this study aims to elucidate the mechanisms underlying this relationship.

Organizational identification is the psychological process through which individuals establish emotional and value-based connections with their employing organization (30, 31). It reflects the degree to which employees cognitively, affectively, and behaviorally align with organizational goals and values (32, 33). Under conditions of high risk and uncertainty, organizational identification is considered a crucial psychological defense mechanism, providing employees with a stable sense of belonging and meaning, thereby mitigating the psychological threats posed by job insecurity (34, 35). Social identity theory posits that strong organizational identification integrates the self-concept with the organization, fostering loyalty and proactive engagement when facing external pressures (36, 37). For CSSD nurses, management support, resource allocation, equitable policies, and leadership care during in the post-COVID-19 period can enhance organizational identification at the cognitive level (38, 39). In this context, organizational identification may serve as a psychological mediator that transforms job insecurity into positive professional motivation, alleviating the negative impact of insecurity on work passion. For example, nurses who strongly identify with their organization’s mission and perceive social recognition of their work may reinterpret insecurity as professional responsibility, thereby fueling intrinsic work passion.

Previous organizational psychology and occupational behavior research predominantly employs variable-centered approaches, using variance and correlation analyses to reveal overall relationships and pathways among variables (40). However, this approach assumes homogeneity within the sample and neglects subgroup differences and latent classification structures. Recently, individual-centered methods have gained traction in psychology, particularly for exploring differential patterns in emotion, motivation, and identification (41, 42). The individual-centered perspective emphasizes identifying combinations of key variables at the individual level and employs latent profile analysis to reveal underlying psychological typologies, offering a more nuanced reflection of psychological heterogeneity and dynamic response patterns in the context of public health emergencies and their aftermath. This study integrates variable-centered and individual-centered dual perspectives to build a comprehensive model that captures both overall trends and individual differences. Variable-centered analysis tests the general relationships and mediation among job insecurity, organizational identification, and work passion; individual-centered analysis identifies nurse subgroups characterized by distinct matching patterns of job insecurity and organizational identification, facilitating the identification of high-risk psychological groups and targeted interventions.

Focusing on CSSD nurses in the post-COVID-19 context, this study aims to explore the association between job insecurity and work passion and the mediating role of organizational identification from both variable-centered and individual-centered viewpoints. The research addresses three core questions: (1) Does job insecurity significantly affect nurses’ work passion? (2) Does organizational identification mediate this relationship? (3) Are there latent subgroups of nurses with distinct psychological profiles of job insecurity and organizational identification? This study’s innovations lie in three aspects: First, targeting a unique population, it fills a gap in COVID-19-related and post-pandemic psychological research on CSSD nurses. Second, it proposes an integrative theoretical model combining social identity theory and positive motivation theory to reveal nurses’ psychological mechanisms through psychological safety and occupational passion pathways. Third, its methodological design employs advanced dual-analysis strategies, offering multidimensional perspectives on occupational psychology under crisis conditions. Practically, the findings aid in identifying high-risk psychological groups and provide hospital management with scientific bases for developing differentiated psychological support and organizational incentive strategies, thereby fostering a more resilient and dynamic nursing workforce in the post-pandemic era.

Although the COVID-19 pandemic provides an important contextual backdrop for understanding hospital work environments, it should not be regarded as the sole explanation for the psychological and organizational experiences of CSSD nurses. Because CSSD staff are responsible for cleaning, disinfection, sterilization, and the safe supply of medical instruments, their work is closely connected to infection prevention and patient safety. The pandemic may have amplified these pre-existing responsibilities and pressures, but concerns related to job insecurity, organizational identification, and work engagement may also arise from broader and ongoing occupational conditions, even among nurses without marked prior pandemic-related stress.

## 2 Methods

### 2.1 Participants

#### 2.1.1 Ethical considerations

This study received approval from the Academic Ethics Committee of Chengdu Third People’s Hospital (Approval No.: 2025-S-341) and strictly adhered to the relevant ethical guidelines issued by China’s National Health Commission. All participants were fully informed about the study’s purpose, potential risks, and benefits prior to participation. Electronic informed consent was obtained, emphasizing voluntary participation, the right to withdraw at any time, and guarantees of anonymity and confidentiality. No personally identifiable information was collected in the questionnaire, and data access was limited strictly to the research team. These measures safeguarded participants’ rights and enhanced the scientific rigor and credibility of the study.

#### 2.1.2 Study design

This research employed a cross-sectional design aimed at systematically examining the impact of job insecurity on work passion among CSSD nurses in the post-COVID-19 period, with a focus on the mediating role of organizational identification. Data collection occurred from September and October 2025, representing the post-COVID-19 phase, allowing for assessment of the pandemic’s dynamic psychological and occupational impact on nurses.

#### 2.1.3 Participant recruitment

Convenience sampling was used with a multi-channel recruitment strategy to maximize sample representativeness and response rates. The electronic questionnaire was developed on a professional data collection platform, generating both the survey and an online link. Coordination with the Chengdu Disinfection Association’s Sterile Supply Committee facilitated internal notifications and electronic posters inviting eligible CSSD nurses to participate. Additionally, we contacted CSSD departments in 26 secondary and tertiary hospitals across Sichuan Province, explaining the study’s purpose, procedures, and benefits to department heads to secure their cooperation. Department leaders assisted in distributing the survey link and promoted participation via internal hospital communication platforms.

#### 2.1.4 Inclusion and exclusion criteria

Inclusion criteria ensured sample specificity and homogeneity: (1) Registered nurses currently or previously working during the pandemic in CSSD; (2) Minimum of 6 months of COVID-19-related work experience, such as ward disinfection or isolation area management; (3) Age 18 or older, with fluency in reading and understanding Chinese; (4) Voluntary participation with completed informed consent.

Exclusion criteria included: (1) Non-CSSD nurses or auxiliary staff (e.g., interns); (2) Questionnaires with less than 80% completion or exhibiting patterned responses (e.g., all identical answers); (3) History of severe psychological disorders to avoid ethical risks; (4) Invalid responses identified through data screening (e.g., extremely short response times). These criteria improved internal and external validity and minimized selection bias.

COVID-19-related personal infection history or direct involvement in the management of COVID-19 cases was not used as an inclusion or exclusion criterion in this study, because the study aimed to investigate work-related psychological and organizational characteristics among CSSD nurses in the broader healthcare context rather than the specific effects of individual pandemic exposure.

#### 2.1.5 Minimum sample size

Sample size estimation combined power analysis for SEM and stability considerations for LPA. Based on Cohen (43) guidelines, assuming a medium effect size (*f*^2^ = 0.15), significance level *α* = 0.05, and power 1-*β* = 0.90, G*Power 3.1 software calculated a minimum sample size of 172 participants. Considering LPA complexity and ensuring at least 50 cases per latent class for stability, the total sample size was set to ≥400. To further enhance statistical robustness and model generalizability, the study aimed to recruit no fewer than 430 nurses.

#### 2.1.6 Final sample

A total of 550 questionnaires were distributed, with 529 returned. During data cleaning, 16 incomplete responses (<80% completion), 7 non-CSSD nurses, and 10 questionnaires showing response patterning (e.g., identical or fixed-order answers) were excluded, resulting in 33 invalid responses. The final sample comprised 496 valid responses, with a response efficiency of 93.76%. Among participants, 164 were male nurses (33.1%) and 332 were female nurses (66.9%). Detailed demographic characteristics are presented in Table 1.

**Table 1:** Demographic information of the study sample.

Variables	Items	Number (N)	Frequency (%)
Gender	Male	164	33.10%
	Female	332	66.90%
Educational level	Secondary school/junior college	124	25.00%
	Undergraduate college	294	59.30%
	Master degree or above	78	15.70%
Place of residence	Cities	387	78.00%
	The countryside	109	22.00%
Marital status	Get divorced	22	4.40%
	Widowed spouse	2	0.40%
	Unmarried	130	26.20%
	Married	342	69.00%
Monthly income level	3,000 RMB or less	93	18.80%
	3,001–5,000 RMB	134	27.00%
	5,001–8,000 RMB	177	35.70%
	8,001 RMB and above	92	18.50%
Length of service	3 years or less	124	25.00%
	3 to 5 years	155	31.30%
	6 to 10 years	148	29.80%
	11 years or more	69	13.90%
Whether to experience COVID-19 on the frontlines of any work.	Yes	374	75.40%
	No	122	24.60%
Age	34.18 ± 6.627		

### 2.2 Measurement instruments

#### 2.2.1 Job insecurity scale

Job insecurity was measured using the scale developed by Vander Elst et al. (44), validated across five European countries. This unidimensional scale consists of 4 items, such as “After COVID-19, do you agree that you are likely to lose your job soon?” It has been widely applied in Chinese populations, including COVID-19 patients (45) and coal miners (46). The scale was translated into Chinese version by Zhang et al. (47), and its cultural adaptability and reliability were verified. Responses were recorded on a 5-point Likert scale ranging from 1 = strongly disagree to 5 = strongly agree. Total scores ranged from 4 to 20, with higher scores indicating greater job insecurity. In this study, the scale demonstrated good internal consistency (Cronbach’s *α* = 0.890).

#### 2.2.2 Organizational identification scale

Organizational identification was assessed with the scale developed by Mael and Ashforth (48), a unidimensional instrument containing 6 items, for example, “When someone criticizes your work unit, it feels like a personal insult.” This scale has been widely used in Chinese samples, including teachers (49) and nurses (50), confirming its cultural adaptability and reliability. The scale was translated into Chinese version by Liu et al. (51) using the back-translation method, and the reliability of the scale was tested in 806 employees. Responses used a 5-point Likert scale from 1 = strongly disagree to 5 = strongly agree. Scores range from 6 to 30, with higher scores reflecting stronger organizational identification. The scale showed good internal consistency in this study (Cronbach’s *α* = 0.822).

#### 2.2.3 Work passion scale

Work passion was measured using the 21-item scale developed by Smith et al. (52), comprising two dimensions: harmonious passion and obsessive passion. This scale has been widely applied in Chinese populations, including workers (52) and healthcare professionals (53), demonstrating good cultural validity. Participants responded on a 5-point Likert scale from 1 = strongly disagree to 5 = strongly agree. Total scores range from 21 to 105, with higher scores indicating stronger work passion. In this study, the scale showed good internal consistency (Cronbach’s *α* = 0.904).

### 2.3 Statistical analysis

Preliminary data processing and descriptive statistics were completed using SPSS 27.0, while latent profile analysis was conducted with Mplus 8.3. Statistical significance was set at two-tailed *p* < 0.05. Robustness was ensured by bootstrap resampling (5,000 iterations) to calculate confidence intervals. Descriptive statistics included means, standard deviations, skewness, and kurtosis. Pearson correlation analyses assessed variable associations to screen for multicollinearity. Reliability was examined using Cronbach’s alpha (*α* > 0.80 indicating good internal consistency). Harman’s single-factor test was used to detect common method bias; variance explained by the first factor below 40% was considered acceptable. Variable-centered analysis employed path analysis via SEM to examine the direct effects of job insecurity on work passion (harmonious and obsessive passion), while the mediating role of organizational identification was tested using PROCESS Macro Model 4. Person-centered analysis applied LPA to identify heterogeneous job insecurity and organizational identification subgroups. Model selection was based on Bayesian Information Criterion (BIC), Akaike Information Criterion (AIC), sample-size adjusted BIC (aBIC), and entropy (>0.80). Bootstrap likelihood ratio tests (BLRT) confirmed profile distinctions.

## 3 Results

### 3.1 Common method bias

Based on previous research, this study employed confidentiality and anonymity during data collection to minimize common method bias (54). To further control for its impact, Harman’s single-factor test was conducted. An unrotated principal component analysis of all measured items identified six factors with eigenvalues greater than 1. The first factor accounted for 27.344% of the total variance, which is below the 40% threshold. Thus, common method bias was not a concern in this study.

### 3.2 Descriptive statistics and correlation analysis

Table 2 presents descriptive statistics and correlations for job insecurity, organizational identification, and work passion. The mean score for job insecurity among CSSD nurses was 2.820 (SD = 0.841), indicating a moderately high level. Organizational identification averaged 3.097 (SD = 0.775), suggesting relatively strong identification with their organization. Work passion had a mean of 3.164 (SD = 0.648), also at a moderately high level. According to Kline (55), skewness values ranged from −0.154 to 0.091 and kurtosis from −0.735 to −0.405, indicating approximate normality of the variables. Correlation analysis revealed a strong significant strong negative relationship between job insecurity and organizational identification (*r* = −0.607, *p* < 0.001), a moderate significant negative correlation between job insecurity and work passion (*r* = −0.448, *p* < 0.001), and a strong significant positive correlation between organizational identification and work passion (*r* = 0.533, *p* < 0.001).

**Table 2:** Descriptive statistics and correlation analyses of core variables.

Variables	*M*	SD	Skewness	Kurtosis	1	2	3
1. Job insecurity	2.820	0.841	0.091	−0.654	1		
2. Organizational identification	3.097	0.775	−0.144	−0.735	−0.607***	1	
3. Work passion	3.164	0.648	−0.154	−0.405	−0.448***	0.533***	1

***p < 0.001.

### 3.3 Mediation analysis of organizational identification

**Figure 1:**
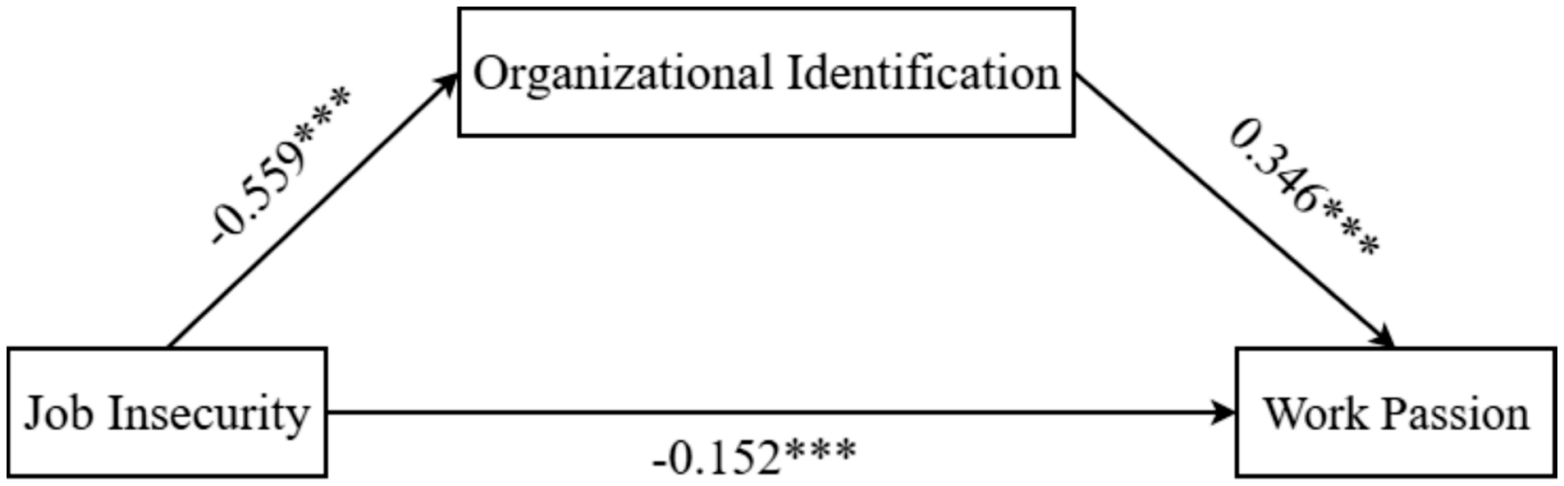
Map of path coefficients identified by the organization.

This study examined the mediating role of organizational identification using job insecurity as an independent variable, organizational identification as a mediating variable, work passion as a dependent variable and demographic information such as gender, age, education, and place of residence as control variables. Mediation analysis was conducted using PROCESS Macro Model 4 with 5,000 bootstrap resamples for confidence intervals (56). Results indicated that job insecurity significantly negatively predicted organizational identification (*β* = −0.559, *p* < 0.001, 95% CI [−0.624, −0.495]); job insecurity also negatively predicted work passion (*β* = −0.152, p < 0.001, 95% CI [−0.223, −0.088]); and organizational identification positively predicted work passion (*β* = 0.346, p < 0.001, 95% CI [0.268, 0.423]), as shown in Table 3 and Figure 1.

**Table 3:** Regression coefficients for organizational identification.

Model	Regression equation		Overall fit index			Regression coefficient			
	Outcome variables	Predictive variables	*R*	*R^2^*	*F*	*β*	*t*	LLCI	ULCI
Model 1	Organizational identification	Job insecurity	0.607	0.369	288.763***	−0.559	−16.993***	−0.624	−0.495
Model 2	Work passion	Job insecurity	0.555	0.308	109.901***	−0.152	−4.173***	−0.223	−0.080
		Organizational identification				0.346	8.767***	0.268	0.423

The mediation effect is summarized in Table 4. Job insecurity’s total negative effect on work passion was −0.345, with 55.9% of this effect explained by organizational identification’s mediation. Specifically, the direct effect accounted for 44.1%. The indirect path via organizational identification was significant (*β* = −0.193, SE = 0.028, 95% CI [−0.249, −0.139]). Therefore, organizational identification partially mediated the relationship between job insecurity and work passion. Job insecurity undermined nurses’ work passion both directly and indirectly by reducing organizational identification.

**Table 4:** Effect decomposition of mediating effects of organizational identification.

Decomposition of effects	*β*	SE	LLCI	ULCI	Proportion of effect
Total effect	−0.345	0.031	−0.448	−0.406	100.0%
Direct effect	−0.152	0.036	−0.223	−0.080	44.1%
Indirect effect	−0.193	0.028	−0.249	−0.139	55.9%

### 3.4 Latent profile analysis

To explore heterogeneity in job insecurity and organizational identification among CSSD nurses, LPA was performed on each item of the two constructs. Models with one to five latent profiles were evaluated using AIC, BIC, adjusted BIC, and entropy values, as shown in Table 5.

**Table 5:** Fit indicators for latent profiles of 1–5 profile of job insecurity and organizational identification.

Profile	AIC	BIC	aBIC	Entropy	LMR (p)	BLRT (p)	Proportions of potential subgroups
1	15634.774	15718.905	15655.425	–	–	–	–
2	14784.835	14915.239	14816.844	0.813	<0.001	<0.001	59.0%/41.0%
3	14570.912	14747.588	14614.279	0.834	<0.001	<0.001	55.0%/17.4%/27.6%
4	14451.004	14673.953	14505.729	0.871	0.115	<0.001	16.4%/4.5%/50.7%/28.4%
5	14350.571	14619.792	14416.654	0.891	0.225	<0.001	2.6%/16.5%/48.2%/28.4%/4.3%

As the number of profiles increased, AIC, BIC, and aBIC values decreased, indicating improved model fit. However, selecting the optimal model also considered interpretability, statistical significance, and profile size and stability. Compared to the one-profile model, the two-profile model showed significant improvement (LMR and BLRT *p* < 0.001), indicating sample heterogeneity. The three-profile model further improved fit significantly (LMR and BLRT *p* < 0.001) with high classification accuracy (entropy = 0.834). Although four- and five-profile models showed slight improvements in fit indices, their LMR tests were not significant (four-profile *p* = 0.115; five-profile *p* = 0.225), suggesting no statistically meaningful improvement. Moreover, the smallest classes in these models represented only 4.5 and 2.6% of the sample, respectively, raising concerns about stability and interpretability. Considering all criteria, the three-profile model was selected as optimal due to its superior statistical performance and theoretical meaningfulness.

**Figure 2:**
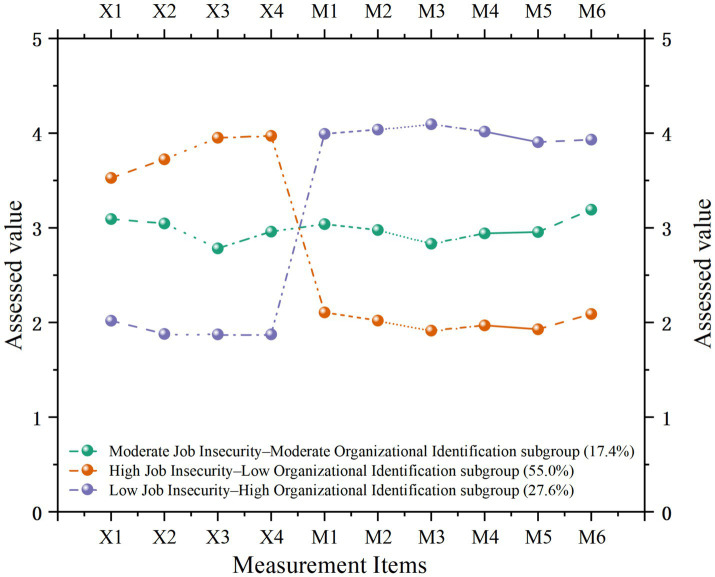
Item-response profiles of the three latent subgroups based on job insecurity and organizational identification. X1–X4 represent the four items of the Job Insecurity Scale, and M1–M6 represent the six items of the Organizational Identification Scale.

Using Origin 2021 software, the item-response patterns of the three-profile solution were plotted in Figure 2. To improve interpretability, the indicators are labeled as X1–X4 for the four job insecurity items and M1–M6 for the six organizational identification items. The three latent profiles accounted for 55.0, 17.4, and 27.6% of the sample, respectively. These profiles represent distinct patterns of job insecurity and organizational identification, reflecting differences in nurses’ perceptions of organizational stability and belonging.

The High Job Insecurity–Low Organizational Identification subgroup (55.0%) reported elevated concerns about job stability and low emotional attachment or value alignment with their organization. Members of this group commonly experienced occupational anxiety and disengagement, showing low work passion. The Moderate Job Insecurity–Moderate Organizational Identification subgroup (17.4%) demonstrated psychological adaptability to uncertainty, maintaining balanced perceptions of risk and moderate organizational attachment. The Low Job Insecurity–High Organizational Identification subgroup (27.6%) perceived strong job stability and security, with high alignment to organizational goals, culture, and values.

### 3.5 One-way ANOVA of work passion across latent subgroups

To assess differences in work passion among latent subgroups, the three profiles were treated as independent variables and work passion as the dependent variable in a one-way ANOVA. Results showed that nurses in the High Job Insecurity–Low Organizational Identification group reported significantly lower work passion (*M* = 2.644, SD = 0.674) compared to the Moderate group (*M* = 3.052, SD = 0.445) and the Low Job Insecurity–High Organizational Identification group (*M* = 3.700, SD = 0.623). The Low Job Insecurity–High Organizational Identification subgroup had the highest work passion. These differences were statistically significant [*F*(2, 493) = 110.572, *p* < 0.001, *η*^2^ = 0.310].

## 4 Discussion

### 4.1 Variable-centered perspective

This study used a variable-centered approach to confirm the direct negative effect of job insecurity on work passion, aligning well with the core predictions of Conservation of Resources (COR) theory. According to Hobfoll’s COR theory, individuals strive to obtain, retain, and protect resources they value (57). When facing actual or threatened resource loss, people experience significant psychological stress, which can lead to negative attitudes and behaviors (58). Job insecurity, as a subjective perception of threats to career continuity and stability, essentially represents a potential loss of core professional resources.

In the unique context of the COVID-19 pandemic, CSSD nurses not only faced physical threats from infection risk but also endured uncertainties from job reassignments, increased workload, and organizational changes. This ongoing threat to their resources drained their psychological reserves, weakening their ability to invest emotional and cognitive energy in their work, ultimately resulting in reduced work passion. Our findings should not be interpreted as indicating that the observed latent profiles were solely shaped by COVID-19-related experiences. Rather, the pandemic may be viewed as a contextual factor that potentially intensified pre-existing workplace demands in the CSSD. Even nurses without substantial prior pandemic-related pressure may still experience job insecurity or reduced organizational identification due to the inherent characteristics of CSSD work, including high responsibility for infection control, repetitive high-standard procedures, workload demands, and reliance on organizational coordination and support.

It is worth noting that the association between job insecurity and work passion in this study was moderate in strength. This finding echoes previous studies on clinical nurses but extends the focus to CSSD nurses—a group playing a key role in pandemic control but often overlooked in research. CSSD nurses have unique job characteristics: although they are not directly involved in patient care, their frequent contact with contaminated medical instruments and strong chemical disinfectants, combined with a closed, repetitive work environment, may heighten burnout risk. By validating the job insecurity–work passion link in this specific group, this study fills a gap in the literature.

Furthermore, this study confirmed that organizational identification partially mediates the relationship between job insecurity and work passion. From a social identity theory perspective, organizational identification reflects the degree to which individuals incorporate their membership in the organization into their self-concept, forming a core psychological bond with the organization (36). When nurses feel high job insecurity, they may doubt the organization’s capacity to protect and support them, leading to psychological distancing and reduced identification with organizational goals and values. This decline in identification undermines their view of work as an extension of self and a source of meaning, thereby decreasing work passion.

The mediating role of organizational identification can also be understood through cognitive reframing. Nurses with strong organizational identification tend to internalize organizational successes and challenges as personal honors and responsibilities (59). Even amid external threats, they are more likely to reinterpret uncertainty as a shared challenge rather than an individual career crisis. This reframing helps protect and even boost their work passion. Conversely, nurses with low organizational identification lack this psychological buffer, making it easier for job insecurity to translate into negative emotions and withdrawal behaviors. The mediation analysis offers clear guidance for hospital management: when completely eliminating job insecurity is unrealistic, strengthening nurses’ organizational identification can effectively buffer its harmful effects on work passion.

### 4.2 Person-centered perspective

This study identified three distinct latent subgroups within CSSD nurses based on job insecurity and organizational identification levels, revealing significant psychological heterogeneity beyond the assumption of homogeneity in traditional variable-centered analyses. The three subgroups were: High Job Insecurity–Low Organizational Identification (55.0%), Moderate Job Insecurity–Moderate Organizational Identification (17.4%), and Low Job Insecurity–High Organizational Identification (27.6%), showing a systematic inverse matching pattern between the two variables.

The first group, High Job Insecurity–Low Organizational Identification, was the largest. Over half of the nurses experienced a dual psychological burden of high job insecurity and low organizational belonging, potentially resulting in emotional exhaustion, reduced work engagement, increased turnover intentions, and stalled career development. These nurses lack organizational support and face persistent worries about their professional future, trapped in a vicious cycle of resource depletion with limited replenishment. This subgroup should be prioritized for psychological interventions and organizational support.

The second group, Moderate Job Insecurity–Moderate Organizational Identification, was the smallest but represents a balanced yet somewhat conflicted psychological state. These nurses remain alert to job uncertainties but have not reached overwhelming anxiety levels; their organizational identification is moderate but unstable. This group may be in a transitional psychological phase with considerable plasticity. Positive organizational interventions could shift them toward the Low Insecurity–High Identification profile, while negative changes might push them toward the High Insecurity–Low Identification profile. Identifying and monitoring this subgroup is valuable for preventive interventions.

The third group, Low Job Insecurity–High Organizational Identification, exhibited the most positive psychological traits and the highest work passion. These nurses are confident in job stability and deeply emotionally connected to the organization’s goals, culture, and values. High organizational identification provides a stable sense of meaning and social support, while low job insecurity frees psychological resources for active work engagement. This healthy resource configuration allows them to experience harmonious passion, viewing work as self-actualization and value creation. Their presence shows that positive psychological states remain achievable despite the widespread pressures of COVID-19, offering a benchmark for intervention strategies.

Notably, the observed inverse matching between job insecurity and organizational identification suggests a bidirectional reinforcing relationship: high job insecurity may erode trust in the organization’s protective capacity, lowering identification, while low identification may reduce perceived organizational support, worsening job insecurity. Breaking this negative cycle requires simultaneous efforts to reduce insecurity and enhance identification; focusing on only one may limit effectiveness.

The person-centered approach’s strength lies in capturing overall variable combinations rather than just average relationships. Traditional variable-centered analyses reveal a negative correlation between job insecurity and organizational identification but cannot show specific individual patterns or proportions. Latent profile analysis fills this gap by identifying subgroups with distinct psychological profiles, guiding precision interventions. This methodological innovation is important for nursing psychology, encouraging future research to integrate both variable-centered and person-centered perspectives.

Importantly, the present study was not restricted to nurses who had suffered from COVID-19 or directly managed COVID-19 cases. This was because CSSD nurses may experience substantial occupational demands even without such direct exposure, given their ongoing responsibilities in disinfection, sterilization, infection prevention, and instrument supply within the hospital system.

### 4.3 Practical and clinical implications

The findings provide empirical support for hospital administrators and nursing leaders to develop targeted psychological support strategies. First, given organizational identification’s key mediating role between job insecurity and work passion, hospitals should prioritize strengthening nurses’ organizational identification to enhance workforce stability and engagement. This can be achieved by cultivating organizational culture, clearly communicating mission and values to help nurses see the connection between their work and organizational goals; implementing fair and transparent HR policies, especially during crises, with timely and accurate communication to reduce information asymmetry and insecurity; and promoting participatory management, giving nurses a voice in decisions affecting their work conditions to boost psychological ownership.

Second, the three latent subgroups identified through latent profile analysis suggest a tiered intervention approach. For the largest High Insecurity–Low Identification group, interventions should simultaneously reduce job insecurity through career planning, skill training, and job security assurances, while increasing organizational identification via leadership support, team-building, and internalizing organizational values. For the Moderate group, focus on prevention by monitoring psychological states and providing early support to avoid decline. For the Low Insecurity–High Identification group, management should maintain and reinforce their positive state and leverage them as role models to spread positive influence through peer support networks.

This study highlights the vital role of nurse managers and nursing leaders in attending to nurses’ psychological states daily. Job insecurity and organizational identification are dynamic, influenced continuously by organizational environment and management behaviors. Nurse managers, as frontline supervisors, should be trained to recognize signs of psychological distress and provide basic psychological support. Additionally, HR departments might integrate psychological capital assessments into routine health checks to identify at-risk individuals early and offer tailored assistance.

These findings have forward-looking significance for post-pandemic nursing workforce management. The psychological impact of COVID-19 on healthcare workers may be long-lasting. Research shows crisis-related psychological effects can persist well beyond the event, manifesting as PTSD symptoms, burnout, or chronic job insecurity. Hospital leaders should not neglect nurses’ mental health as the pandemic subsides but incorporate ongoing psychological support into routine HR practices.

### 4.4 Limitations and future research directions

Despite these findings, several limitations exist. This study’s cross-sectional design can only reveal associations, not causal relationships. Although theory guided the hypothesized causal path from job insecurity to organizational identification to work passion, reverse or reciprocal causality cannot be ruled out. For example, nurses with low work passion might evaluate their work environment more negatively and report higher job insecurity. Future longitudinal studies measuring variables at multiple time points are needed to clarify temporal and causal directions.

The sample was drawn from secondary and tertiary hospitals in Sichuan Province, China, potentially limiting generalizability. Nurses in different regions and types of hospitals may experience different work environments and organizational cultures, affecting psychological responses. Future research should include wider geographic and institutional samples to enhance external validity. Cross-cultural comparisons would also be valuable to explore cultural boundary conditions of the findings.

Data were collected via self-report questionnaires, which may introduce social desirability and common method biases. Although Harman’s single-factor test suggested minimal common method bias, future studies can strengthen robustness by using multi-source data such as supervisor or peer ratings and objective performance indicators. Qualitative methods like in-depth interviews could further enrich understanding of subjective experiences and meaning-making around job insecurity and organizational identification.

This study focused mainly on the mediating role of organizational identification, but other important mediators or moderators may exist. Organizational factors such as leadership support, coworker relationships, and job autonomy, as well as individual factors like resilience and self-efficacy, might influence the job insecurity–work passion link. Future research could develop more complex mediation and moderated mediation models to fully elucidate mechanisms and boundary conditions.

Finally, data were collected during the later stages of the COVID-19 pandemic; nurses’ psychological states may differ from those at the peak. Acute stress responses during the crisis and chronic stress adaptation afterward likely have distinct psychological profiles and effects. Future longitudinal designs tracking psychological trajectories across pandemic phases would provide a comprehensive understanding of crisis impacts on nurse mental health.

## 5 Conclusion

In the post–COVID-19 context, CSSD nurses continue to work under persistent uncertainty and heightened infection-control demands, making job insecurity a salient occupational stressor with potential implications for workforce stability and hospital preparedness. Using a dual-perspective framework, this study provides evidence that job insecurity is negatively associated with work passion among CSSD nurses, and that organizational identification partially explains this association. In other words, higher perceived insecurity is linked to lower organizational identification, which in turn corresponds to reduced work passion, highlighting organizational identification as an important psychological resource in sustaining positive work motivation.

Beyond average effects, latent profile analysis revealed meaningful heterogeneity within this population. Three distinct profiles—High Job Insecurity–Low Organizational Identification, Moderate Job Insecurity–Moderate Organizational Identification, and Low Job Insecurity–High Organizational Identification—showed graded differences in work passion, with the low-insecurity/high-identification group reporting the highest passion. These findings underscore the value of stratified management approaches: alongside efforts to reduce employment uncertainty, hospitals should prioritize interventions that strengthen organizational identification through transparent communication, fair and supportive management practices, and recognition of CSSD contributions to infection prevention and control.

The study advances understanding of the psychological mechanisms linking job insecurity to work passion among an often overlooked but essential nursing workforce. Future longitudinal and multi-source studies are warranted to clarify causal directions and to test targeted interventions designed to enhance organizational identification and protect work passion in the long-term aftermath of public health emergencies.

## Data Availability

The raw data supporting the conclusions of this article will be made available by the authors, without undue reservation.
